# Validations of Blood Pressure Measuring Devices Using Recognized Protocols

**DOI:** 10.3390/jpm13010009

**Published:** 2022-12-21

**Authors:** Victoria Mazoteras-Pardo, Sagrario Gómez-Cantarino, Miguel Ramírez-Jiménez, Emmanuel Navarro-Flores, María Idoia Ugarte-Gurrutxaga

**Affiliations:** 1 Research Group “ENDOCU”, Department of Nursing, Physiotherapy and Occupational Therapy, Faculty of Physiotherapy and Nursing of Toledo, University of Castilla-La Mancha, 45071 Toledo, Spain; 2 Faculty of Education REDAFLED, University of Valladolid, 42004 Soria, Spain; 3 Frailty Research Organized Group (FROG), Department of Nursing, Faculty of Nursing and Podiatry, University of Valencia, 46001 Valencia, Spain

**Keywords:** blood pressure, hypertension, blood pressure monitoring, validation

## Abstract

Preventing, diagnosing, and controlling high blood pressure is a global health priority. The self-measurement of blood pressure is therefore fundamental and should be done with devices validated by recognized protocols, although most are not. The most widely used and current protocols are the 2010 European Society of Hypertension (ESH) revision and the 2018 Association for the Advancement of Medical Instrumentation (AAMI)/ ESH/ the International Organization for Standardization (ISO) universal standard, respectively. The aim of this study was to find out which blood pressure measuring devices have been adequately validated by the above protocols. A narrative review of blood pressure device validations was conducted by searching the PubMed database. From 52 records identified, 37 studies were included. Most validations follow the 2010 revision and only six follow the 2018 protocol, which is more demanding. Almost all validated sphygmomanometers are automated oscillometric sphygmomanometers in the general population. Wrist devices and devices combining new technologies are also validated, as well as in specific populations, such as the obese, pregnant women, or children. There is sufficient evidence to confirm that the universal AAMI/ ESH/ISO standard is considered the protocol of the century. However, it is necessary to increase the number of validations following it and, above all, validations of the new technologies that are invading the current market.

## 1. Introduction

After obesity, arterial hypertension (HBP) is the second most common cause of cardiovascular disease (CVD) [1]. If we add that CVD is the leading cause of morbidity and mortality [2,3], we can conclude that AHT carries a high risk of CV morbidity and mortality [4,5,6,7].

Therefore, one of the main objectives of health systems is to identify people with AHT and ensure that they have good control of their blood pressure (BP), since the higher the blood pressure levels, the greater the risk and morbidity and mortality from CV events [8,9,10]. Thus, preventing, diagnosing, treating, and controlling hypertension is a global health priority [4,5,7,9,10,11,12,13,14].

The detection and diagnosis of hypertension must be done by measuring BP. BP measurement is considered as one of the most frequently performed procedures at the clinical level, in primary or specialized care [15].

The first time BP was recorded in a consultation was in 1896, and since the end of the last century, out-of-office measurement has been established in two versions: ambulatory BP monitoring (ABPM) and home blood pressure monitoring (HBPM). The major advantage of these two methods is that by allowing BP to be measured on multiple occasions outside the healthcare environment, it provides a more reliable BP reading and has a higher prognostic value than doing it in the office [16,17,18].

If we focus on HBPM, it has become a very beneficial and increasingly used simple procedure, for highly consistent reasons [19,20,21]. The subject’s self-measurements are very useful for the monitoring of HBP, since it is estimated that about 80–90% of the doubts in the diagnosis and control of this pathology can be solved with this procedure, but, logically, these benefits can be obtained if the HBPM is done properly and with validated devices [15,17,22,23].

With regard to blood pressure validations, in the last 30 years, there have been several protocols for this purpose, such as the British Society of Hypertension (BSH) [24] protocol, the Association for the Advancement of Medical Instrumentation (AAMI) protocol, [25] and the international protocol published by the European Society of Hypertension (ESH) [26] and its review [27]. The latter [26,27] were the most current and widely used, but given the need to increase the validity of these devices, in 2018, experts from the AAMI, ESH, and the International Organization for Standardization (ISO) agreed to develop a universal standard for their validation. Today, it is considered as the single universal standard and replaces all other previous standards/protocols [25,28,29].

Given the relevance of HBPM and the need for increased monitoring of BP devices, according to the literature, the results of the present study will mainly serve to find out which devices can be validly used by subjects to measure and monitor their blood pressure, according to the most used and/or current protocols [27,29].

## 2. Materials and Methods

### 2.1. Study Design

A narrative review was carried out following the applicable recommendations of the Scale for the Assessment of Narrative Review Articles (SANRA) [30]. This scale contains six items to assess the quality of narrative review articles. It can be found in the Supplementary Material. The aim of this review was to update the data of the devices available on the market that are valid for measuring blood pressure since there are no recent previous systematic reviews on the issue.

### 2.2. Search Strategy

The database search was carried out during November–January 2021. Pubmed was the database used for this process.

The advanced search strategy was as follows, combining the terms with the Boolean and grouping operators that follow: (European Society of Hypertension [Title]) OR AAMI/ESH/ISO[Title]) AND validation [Title].

As for the search filters, only one of the publication dates is applied: Last 5 years.

The search strategy has been filtered by title because, by regulation, all validations following the “the revised 2010 European Society of Hypertension international protocol” and “the 2018 AAMI/ESH/ISO universal standard” should be named in a standardized way and, consequently, the titles of these publications should also be named in the same way.

### 2.3. Selection Criteria

Inclusion criteria comprised blood pressure device validation studies that followed the 2010 European Society of Hypertension international protocol review and/or the 2018 AAMI/ESH/ISO universal standard, conducted within the last 5 years.

Exclusion criteria included studies with a publication date prior to 2017, and those that were not device validations or did not follow the revised 2010 European Society of Hypertension international protocol and/or the 2018 AAMI/ESH/ISO universal standard.

### 2.4. Data Extraction

The data obtained were divided according to the validation protocol used. The common methodology governing the validation conditions was recorded, such as sample size, blood pressure range, and other variables, in accordance with the protocol used.

Study characteristics were also recorded, such as citation year and place of validation, types of devices used, population characteristics and origin, and validation conclusions.

Additional information was provided where necessary.

### 2.5. Flow Diagram

**Figure 1:**
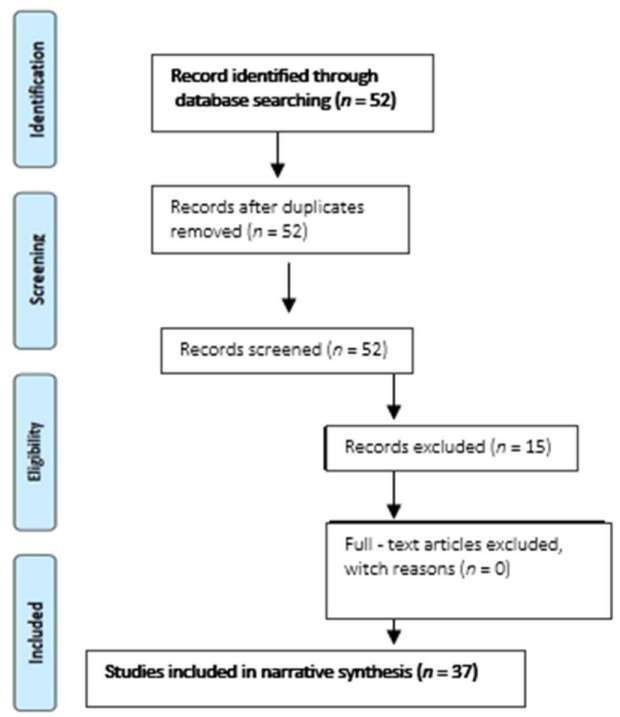
Flow diagram of the narrative review in the Pubmed database.

From 52 records identified through the searching process, 15 were removed due to exclusion, and finally, 37 studies were included in narrative synthesis (Figure 1).

15 studies were excluded for the following reasons:

− 7 articles discussed the validation standard itself and/or revisions of the validation standard
− 2 studies commented with the proper use of validation protocols
− 4 publications were corrections of previously included articles.
− 1 paper was about validation but only followed the ESH 2002 protocol.
− 1 study did not analyze validity but reproducibility.

## 3. Results

### 3.1. Characteristics of the Validation Protocols

The studies of the last five years on blood pressure measuring devices mainly apply two validation protocols for BP devices, which are the review of the international ESH protocol [27] and the international universal standard AAMI/ESH/ISO 81160-2:2018 [29], the latter being the most recent.

Thus, 31 articles following the ESH review [27] and six based on the AAMI/ESH/ISO universal standard [29] are found in the literature. The methodology of these studies has common characteristics, depending on the validation protocol they follow (Table 1). The characteristics of the sample, the sample recruitment criteria, the blood pressure measurement method, or the analyses required to validate the device in question will differ.

Both protocols use a similar sequential BP measurement procedure by alternating two devices (reference and test). Validated devices are already used as a reference, but the 2010 ESH prefers these to be two mercury sphygmomanometers with stethoscopes and the AAMI/ESH/ISO standard indicates that the reference can be as stated above, but can also be non-mercury sphygmomanometers, aneroid manometers, or other types. The measurement conditions are similar, where the subject being measured must remain relaxed and calm in a certain position. Also, the human validation equipment is almost identical, and it has a similar tolerable BP measurement error. In both protocols, Bland–Altman charts are required.

On the other hand, they vary substantially in the range of BP recruitment, sample characteristics and size, and validation criteria.

For a device to pass the ESH [27], it must pass two phases. To pass the first phase, two conditions must be met: a minimum of 65, 81, and 93 comparisons falling within 5, 10, and 15 mm/Hg, respectively; a minimum of two of the following three requirements: 73, 87, and 96 differences must be within the category of 5, 10, and 15 mm/Hg, respectively. In the second phase, a minimum of 24 subjects are required to have two of their three differences in the 5 mm/Hg category, and a maximum of three individuals with the three differences greater than 5 mm/Hg is allowed.

To pass the universal standard [29], the device must also pass two phases. In the first phase, the mean BP difference must be 5 mm/hg or less, and its standard deviations 8 mm/hg or less for SBP and DBP. In the second phase, the standard deviation of 85 averaged BP differences (test minus reference BP per subject) must be within a threshold defined by the mean test-reference BP difference listed for SBP and DBP.

In terms of specific populations, the standards proposed by the three societies as a whole are more explicit.

Thus, although the ESH [27] protocol improved on previous protocols [24,26,27] by eliminating some validation steps and reducing the sample size, it is simpler to apply than the most recent protocol. The international universal validation protocol (AAMI/ESH/ISO) of 2018 [29] is currently the most complete but more complex than the previous one.

### 3.2. Characteristics of the Studies

All the studies collected have the same quality in terms of study type, as they all follow the same standards and have the same design: prospective cross-sectional observational studies [31].

Having analyzed the common and different aspects in terms of the validation conditions governing the respective protocols (Table 1), we now turn to the rest of the characteristics, which are summarized in Table 2.

## 4. Discussion

We found positive results in most studies, exceeding the protocol in question and validating the test devices for their recommended use, as well as in the population analyzed, with the exception of two studies with four test devices, Omron RS6 (hem-6221-E), Microlife WatchBP O3 [33], Yuwell YE680a, and Cofoe KF-65B [67].

The validated devices are different depending on the measurement method, the area of the body where they are applied, their characteristics, or their function: self-measurement, BP monitoring, or professional use, among others. Only one study has validated a device that uses the auscultatory method to measure BP [66]. Typically, oscillometric upper arm sphygmomanometers are validated as the conventional devices for measuring BP and heart rate (HR). These consist of an automatic monitor with an LCD display and a cuff connected by rubber tubes, with some exceptions, which do not have tubes connecting the pump to the cuff; rather, the device itself is embedded in the cuff [42,47,53,55]. They are usually battery operated and some have various cuff sizes.

The cuff used by most authors in their test device is usually the standard or medium size (22–32 cm), except for some who have tested more than one cuff, such as small (18–22 cm) [65], or large (32–42 cm) [65,66].

There are authors who opt for other wrist devices of the same type [33,35,38,44,50,61] and therefore do not have rubber tubes. These are more applicable to obese subjects because they have a larger arm circumference, giving erroneous measurement results if a standard upper cuff is used [33].

Of the above, many are recommended devices for HBPM but some are designed for ABPM [32,36,46,48,53], while there are few validated sphygmomanometers that are used only in a professional manner [58,59,65,66]. According to the ESH protocol, the validation of ABPM devices is performed only in static conditions and does not require testing in ambulatory conditions.

Among those used for HBPM, other validated devices differ more from the above. The Inbody BPBIO320 [49] and the Inbody BPBIO750 [62] are for public use as well as being automatic right upper arm oscillometric devices, but in this case, it is a kiosk type with a fixed hole for the user to insert their arm to measure their BP. Other devices have also been validated that measure BP oscillometrically and can be connected wirelessly to electronic devices allowing for better visual recording and monitoring [46,51,63]. Other units have similar behavior but are operated directly from an app connected to the oscillometric cuff via Bluetooth [42,47,55]. To this effect, one study tests an app that measures BP without a cuff through fingerprints [68] but it is difficult to follow the validation protocol with it.

Therefore, of all the validations, only one device without a cuff to measure BP is attempted to be validated [68], and the results are unsuccessful.

It should be noted that the use of new technologies for health promotion and disease prevention is booming and, in particular, BP control using these has gained importance, adding additional advantages to HBPM, which results in a more active subject with better adherence and follow-up [68,69,70,71]. Despite this and its widespread use and downloading by society, only six apps are being validated [42,46,47,51,55,63,68], considering that one of them, Qardioarm, has been validated on several occasions, by different teams and in different populations [42,47,55]. This is not a unique case, since it draws our attention to the fact that the Omron RS6 is tested on obese subjects and is found to be invalid by some authors [33] and valid by others [61]. This may be due to the fact that both studies follow the ESH review and do not have specific criteria for vulnerable populations [27].

As for the parameters assessed in the populations, all studies include recruitment SBP and DBP, age (in years), and circumference of the upper arm [32,34,36,37,39,40,41,42,43,45,46,47,48,49,51,52,53,54,55,56,57,58,59,60,62,63,64,65,66,67], wrist [38,40], or both [25,33,44,61] (in mm), depending on the device to be tested. Only one study does not measure any circumference of its participants [68]. BMI is also assessed by some authors [33,34,38,45,46,47,51,55,57,61], although only some of these [47,51,55,61] reflect the weight and height of their participants.

These measured characteristics are generally similar as they all meet the recruitment criteria of the protocols [27,29], except for special populations.

Most of these studies are conducted in the general population, which are healthy adults over 25 years of age for one protocol [27], and 12 years of age for another [29]. In all of them, the number of women recruited tends to be higher than that of men.

Some studies look at other specific populations prone to HBP, such as the obese [33,47,61], children [36,65], diabetics [42], pregnant women [48], or subjects with chronic kidney disease [55]. Furthermore, the difference between the recruited and tested subjects is often greater in these specific populations, as more failures encountered tend to emerge. Most of the reasons for exclusion are full BP ranges. The above are necessary because the results obtained in the general population cannot be extrapolated to specific populations, as they do not follow the same recruitment conditions, requiring a larger sample in most cases. Hence, the universal standard already applies explicit criteria for such populations [29] where even stricter BP control is necessary.

As for the validation team, it also follows the rules of the protocols [27,29], reflected in Table 1, so it usually corresponds to two observers and a supervisor, except for others that have additional explanations, such as teams formed by three licensed physicians [32,48,58], three nurses [47,55], four physicians [49], or three medical technologists [50,59]. All teams were trained in BP measurement.

Although the universal standard is considered the protocol of the century [29], only seven articles have been seen to follow it [56,58,59,62,65,66,68] compared to the large number of studies that follow the ESH [27], almost all of them with positive results [72,73], because although they share conditions, it is less strict (Table 1).

For example, it was recommended to the journals that, from November 2019 onward, they no longer accept articles following the ESH to validate the devices [74] and only accept the most recent protocol [29], so it is considered necessary to increase the monitoring of this type of device, especially their apps, following the same protocol. In addition, a mercury-free sphygmomanometer can be used as the gold standard in this protocol and is used by the ESH.

To conclude this section, it is worth mentioning that one of the main limitations of our study is that we have only used one database, Pubmed. We were guided by a study that analysed optimal database combinations for literature searches and found that “Sixteen percent of the included references (291 articles) were only found in a single database” [75].

## 5. Conclusions

Since 2017, 37 devices have been attempted to be validated to measure BP with reputable protocols, with most obtaining positive results. It is necessary to increase the number of studies according to what is considered the protocol of the century, the universal AAMI/ESH/ISO standard (ISO 81160-2:2018), and especially using new technologies, as few validations have been found on them.

## Figures and Tables

**Table 1:** Characteristics of the main validation protocols.

	ESH Protocol Review 2010	AAMI/ESH/ISO Universal Standard ISO 81160-2:2018
Validation Team	1 independent supervisor, 2 observers trained in BP	1 independent supervisor, ≥ 2 observers trained in BP
Sample	Men and women ≥ 25 years old	Males and females ≥ 12 years old
Sample Size	33 subjects (≥10 men; ≥10 women)	85 subjects (≥30% men; ≥30% women)
Recruitment BP mm/hg	SBP: 10–12 subjects in 3 groups of SBP: 90–129, 130–160, 160–180 DBP: 10–12 subjects in 3 groups of DBP: 40–79,80–100, 100–130	SBP readings: ≥ 5% ≤100; ≥ 5% must be ≥ 160 and ≥ 20% ≥ 140 DBP readings: ≥ 5% ≤ 60; ≥ 5% ≥ 100 and ≥ 20% ≥ 85
Cuff sizes	Not applicable	For test devices with a single cuff: ≥40% subjects must have an arm circumference within the (upper half) of the specified range of use of the cuff; ≥40% (lower half); ≥20% (higher quarter; ≥20% (lower quarter); ≥10% (higher octal); ≥10% (lower octal)
Measurement conditions	Room: Quiet, isolated, comfortable temperature. Subject: Relaxed ≥ 5 min, empty bladder, silent, sitting, feet resting on the floor without crossing their legs, their arm on a flat surface at the level of the heart, palm of the hand upwards.	Idem
Measurement method 9 sequential measurements (2 initial + 7 validation) alternating two devices Same sequential arm	-Gold standard: Two standard mercury sphygmomanometers with nonelectronic stethoscopes (observers) -Test instrument (supervisor) 30–60 s between measurements	-Gold standard: The auscultatory method with dual-head stethoscope (observers). -Test instrument (supervisor) 60 s between measurements The opposite arm simultaneous method can also be used.
Analysis method	3 comparisons per subject = 99 pairs of measurements. Each test measurement is compared with two references (the one before and the one after) and the smallest difference remains. 2 validation phases, based on absolute BP differences within 5, 10, and 15 mm/hg Bland–Altman graphics	3 comparisons per subject = 255 paired BP comparisons Each of the test device measurements is compared against the average of the previous and succeeding reference BP readings. Differences are calculated by subtracting the reference from the test device measurement. 2 validation phases: BP differences ≤ 5 and SD ≤8 mm/hg For individual subjects, the SD of 85 averaged BP differences must be within a threshold defined. Only applicable in samples of 85 subjects Bland–Altman graphics
Exclusion criteria	Arrhythmia; Other problems during the validation Reasons for exclusion reported	Idem
Specialized populations	No particular conditions are indicated Suggests adapting the conditions	Special conditions: Children ≤ 3 years, pregnant; arm circumference ≥ 42 cm

AAMI, Association for Advancement of Medical Instrumentation; BP, blood pressure; cm, centimeters; DBP, diastolic blood pressure; ESH, European Society of Hypertension; ISO, International Organization for Standardization; mm/hg, millimeters of mercury; s, seconds; SBP, systolic blood pressure; SD, standard deviation.

**Table 2:** Comparative table of validation studies.

PUBLICATION	PROTOCOL	BP DEVICES	POPULATION	RESULT
2017. Abou-Dakn et al. [32]	ESH-IP 2010	Gold standard: ERKAMETER 3000 (ERKA, Bad Tölz, Ger-many), mercury sphygmomanometer Test device: TONOPORT VI, oscillometric, ABPM	General population	TONOPORT VI passed
2017. Azaki et al. [33]	ESH-IP 2010	Gold standard: two mercury sphygmomanometers and stethoscope 2 Test devices: OMRON RS6 (HEM-6221-E), automatic oscillometric, HBPM, wrist MICROLIFE WATCHBP O3 (BP 3 MZ1-1), automatic oscillometric office, ABPM, HBBM, arm	Obese population	OMRON RS6, MICROLIFE WATCHBP O3 failed
2017. Chen et al. [34]	ESH-IP 2010	Gold standard: a mercury sphygmomanometer Test device: BPUMP BF1112, automated electronic digital oscillometric, HBPM, arm	General population	BPUMP BF1112 passed
2017. Liu et al. [35]	ESH-IP 2010, BHS protocol; ISO 81060-2:2013 criteria	Gold standard: mercury sphygmomanometers (Yuyue; Yuyue Medical Equipment Co., Ltd., Jiangsu, China) with a double stethoscope Test device: G.LAB MD2200, automated oscillometric, wrist	General population	G.LAB MD2200 passed
2017. Beime et al. [36]	ESH-IP 2010 modified according to KIGGS	Gold standard: ERKAMETER 3000 (ERKA, Bad Tölz, Ger-many), mercury sphygmomanometer with a double stethoscope (ErkaPhon). Test device: CUSTO SCREEN PEDIATRIC, oscillometric, automatic zero balancing, ABPM of children	Child population	CUSTO SCREEN PEDIATRIC passed
2017. Fania et al. [37]	ESH-IP 2010	Gold standard: mercury sphygmomanometer Test device: A&D UM-201 (Tokyo, Japan), automatic oscillometric, office BP, arm	General population	A&D UM-201 passed
2017. Kang et al. [38]	ESH-IP 2010	Gold standard: mercury sphygmomanometer Test device: AVITA BPM17 (AVITA Medical, Taipei, Taiwan), automatic oscillometric digital, HBPM, wrist	General population	AVITA BPM17 passed
2017. Fania et al. [39]	ESH-IP 2010	Gold standard: mercury sphygmomanometer Test device: A&D UM-211 (Tokyo, Japan), automatic oscillometric, office BP measurement, arm	General population	A&D UM-211 passed
2017. Chen Q et al. [40]	ESH-IP 2010	Gold standard: mercury sphygmomanometer Test device: YUWELL YE690A (Danyang, Jiangsu Province, China), automatic digital oscillometric, office BP, HBPM, arm	General population	YUWELL YE690A passed
2017. Grover-Páez et al. [41]	ESH-IP 2010	Gold standard: mercury sphygmomanometer Test device: OMRON HEM-7320-LA (Omron Healthcare Co., Ltd., Kyoto, Japan), automatic intelligent wrap, oscillometric, office BP, HBPM, arm	General population	OMRON HEM-7320-LA passed
2017. Chahine et al. [42]	ESH-IP 2010	Gold standard: mercury sphygmomanometer 2 Test devices: QARDIOARM (San Francisco, CA, USA), automatic oscillometric, HBPM, upper, connected by bluetooth to mobile phones and tablets through the Qardio app OMRON M6 IT COMFORT^®^ HEM-7322U-E (Omron Healthcare Co., Ltd.,Kyoto, Japan), automatic digital oscillometric, HBPM, arm	1: General population 2: Non-insulin-dependent type II diabetic	QARDIOARM, OMRON M6 passed
2018. Chen L. et al. [43]	ESH-IP 2010; BHS Protocol	Gold standard: a mercury sphygmomanometer with Y-tubing dual-head stethoscope Test device: PANGAO PG-800B26 (Shenzhen Pango Electronic Co., Ltd., Shenzhen, China), automatic electronic oscillometric, HBPM, arm	General population	PANGAO PG-800B26 passed
2018. Zhao et al. [44]	ESH-IP 2010; BHS Protocol	Gold standard: a mercury sphygmomanometer Test device: PANGAO PG-800A36 (Shenzhen Pango Electronic Co., Ltd., Shenzhen, China), automatic electronic oscillometric, HBPM, wrist	General population	PANGAO PG 800A36 passed
2018. Kang et al. [45]	ESH-IP 2010	Gold standard: mercury sphygmomanometer Test device: AVITA BPM64 (AVITA Medical, Taipei, Taiwan), an automated electronic digital oscillometric, HBPM, arm	General population	AVITA BPM64 passed
2018. Pereira et al. [46]	ESH-IP 2010	Gold standard: mercury sphygmomanometer Test device: BENEWARE MODEL ABP-021 (Suzhou Beneware Medical Equipment Co., Ltd., Hangzhou, China), automatic oscillometric ABPM, arm This has a built-in USB communication interface enabling connection with a PC operating the dedicated ABPM analysis software.	General population	BENEWARE MODEL ABP-021 passed
2018. Mazoteras-Pardo et al. [47]	ESH-IP 2010	Gold standard: mercury sphygmomanometer. Test device: QARDIOARM (Qardioarm, Atten Electronic Co., Dongguan, China), automatic oscillometric HBPM, arm connected by bluetooth to mobile phones and tablets through the Qardio app	Obese population	QARDIOARM passed
2018. Abou-Dakn et al. [48]	ESH-IP 2010	Gold standard: ERKAMETER 3000 (ERKA, Bad Tölz, Germany), mercury sphygmomanometer Test device: PHYSIO-PORT UP (AR Medizintechnik GmbH & Co.KG, Berlin, Germany), oscillometric ABPM, arm	Pregnant	PHYSIO-PORT UP passed
2019. Kollias et al. [49]	ESH-IP 2010	Gold standard: Two mercury sphygmomanometers (Baumanometer; WA Baum Co., Inc., New York City, NY, USA) with stethoscope (3M Littmann Classic II SE; 3M, St Paul, MN, USA) Test device: INBODY BPBIO320 (InBody, Seoul, Korea), kiosk-type automated oscillometric HBPM, right arm, public spaces. It has a fixed hole to insert the user’s arm, with an implanted single cuff arm.	General population	INBODY BPBIO320 passed
2019. Saito et al. [50]	ESH-IP 2010; ANSI/AAMI/ISO 81060-2:2013 protocol	Gold standard: mercury sphygmomanometer 2 Test devices: OMRON HEM-6232T (Omron Healthcare Co., Ltd., Kyoto, Japan), automatic oscillometric with a sensor for the angle of the forearm, wrist OMRON HEM-6181 (Omron Healthcare Co. Ltd., Kyoto, Japan), automatic oscillometric, wrist	General population	OMRON HEM-6232T, OMRON HEM-6181 passed
2019. Mazoteras-Pardo et al. [51]	ESH-IP 2010	Gold standard: mercury sphygmomanometer Test device: IHEALTH TRACK (KN-550BT; iHealthLabs Europe, Paris, France), automatic oscillometric, arm	General population	IHEALTH TRACK passed
2019. Reshetnik et al. [52]	ESH-IP 2010	Gold standard: ERKAMETER 3000 (ERKA, Bad Tölz, Germany), mercury sphygmomanometer Test device: NIBP2020 UP technology (PAR Medizintechnik GmbH and Co. KG, Berlin, Germany), an electronic board, which must be installed into a host system: BP+ (Uscom Ltd., Sydney, Australia) has been used as a host device, automatic oscillometry, arm	General population	NIBP2020 UP technology passed
2019. Fania et al. [53]	ESH-IP 2010	Gold standard: mercury sphygmomanometer Test device: HINGMED WBP-02A (Hingmed Company Shenzhen, China), automated oscillometric for ABPM 24 h, arm	General population	HINGMED WBP-02A passed
2019. Liu et al. [54]	ESH-IP 2010	Gold standard: mercury sphygmomanometer Test device: TRANSTEK TMB-1776 (Guangdong Transtek Medical Electronics Co., Ltd., Zhongshan, China) an automatic oscillometric, HBPM, arm	General population	TRANSTEK TMB-1776 passed
2019. Mazoteras-Pardo et al. [55]	ESH-IP 2010	Gold standard: Omron M3 Intellisense (Omron Healthcare) Test device: QARDIOARM (Qardioarm, Atten Electronic Co., Dongguan, China), automatic oscillometric, HBPM, upper connected by bluetooth to mobile phones and tablets through the Qardio app	Patients with chronic kidney disease	QARDIOARM passed
2020. Kollias et al. [56]	AAMI/ESH/ISO universal standard (ISO 81160-2:2018)	Gold standard: two mercury sphygmomanometers Test device: INBODY BP170 an automated oscillometric, HBPM, upper	General population	INBODY BP170 passed
2020. Zhang et al. [57]	ESH-IP 2010	Gold standard: mercury sphygmomanometer Test device: HL868ED, automated electronic digital oscillometric, HBPM, office BP, arm	General population	HL868ED passed
2020. Kollias et al. [58]	AAMI/ESH/ISO universal standard (ISO 81160-2:2018)	Gold standard: two mercury sphygmomanometers (Baumanometer; WA Baum Co., Inc., New York, NY, USA) Test device: INBODY BPBIO250, automated oscillometric, office BP, arm	General population	INBODY BPBIO250 passed
2020. Saito et al. [59]	ESH-IP 2010; ANSI/AAMI/ISO 81060-2:2013 protocol	Gold standard: mercury sphygmomanometer Test device: OMRON HBP-1320 (Omron Healthcare Co., Ltd., Kyoto, Japan), upper arm BP device. Both the automatic oscillometric mode and the auscultation mode can be selected. Oscillometric mode has been used.	General population	OMRON HBP-1320 passed
2020. Song et al. [60]	ESH-IP 2010	Gold standard: mercury sphygmomanometer Test device: GLOBALCARE GCE603 (Globalcare Medical Technology CO., LTD., Zhongshan, Guangdong, China), an automatic digital oscillometric, HBPM, arm	General population	GLOBALCARE GCE603 passed
2020. Omar et al. [61]	ESH-IP 2010	Gold standard: Two mercury sphygmomanometers and two non-electronic stethoscopes Test device: OMRON RS6 (HEM-6221-E), automatic digital oscillometric wrist device for HBPM.	Obese population	OMRON RS6 (HEM-6221-E) passed
2021. Ntineri et al. [62]	AAMI/ESH/ISO universal standard (ISO 81160-2:2018)	Gold standard: mercury sphygmomanometer Test device: INBODY BPBIO750 (InBody, Seoul, Korea), kiosk-type automated oscillometric right arm, HBPM, public spaces.	General population	INBODY BPBIO750 passed
2021. Chachine et al. [63]	ESH-IP 2010	Gold standard: mercury sphygmomanometer Test device: PHILIPS DL8760, utomatic oscillometric, HBPM, arm This unit is integrated into the cuff	General population	PHILIPS DL8760 passed
2021. Deutsch et al. [64]	ESH-IP 2010	Gold standard: mercury sphygmomanometer Test device: BEURER BM 28, automatic digital oscillometric, HBPM, arm	General population	BEURER BM 28 passed
2021. Zhang et al. [65]	AAMI/ESH/ISO universal standard (ISO 81160-2:2018)	Gold standard: mercury sphygmomanometer Test device: YUWELL YE900, automatic digital oscillometric, office BP, arm. 3 cuffs	General population and children	YUWELL YE900 passed
2021. Ntineri et al. [66]	AAMI/ESH/ISO universal standard (ISO 81160-2:2018)	Gold standard: mercury sphygmomanometer Test device: INBODY BPBIO210 (InBody, Seoul, Korea), a manual auscultatory mercury-free hybrid, office BP, arm, 2 cuffs	General population	INBODY BPBIO210 passed
2021. Zhang et al. [67]	ESH-IP 2010	Gold standard: mercury sphygmomanometer 3 Test devices: OMRON HEM-7120 (OMRON Healthcare Co., Ltd., China); YUWELL YE680A (Jiangsu Yuwell Medical Equipment Co., Ltd.); COFOE KF-65B (Hunan Cofoe Medical Technology Development Co., Ltd.), automatic oscillometric, HBPM, arm	General population	OMRON HEM-7120 passed YUWELL YE680A and COFOE KF-65B failed
2021. Degott et al. [68]	AAMI/ESH/ISO universal standard (ISO 81160-2:2018) with modifications imposed by cuffless measurements.	Gold standard: A&D UM-101, mercury-free sphygmomanometer (A&D Company, Ltd., Toshima Ku, Tokyo, Japan) Test device: OPTIBP MOBILE APPLICATION (Biospectal SA, Lausanne, CH) installed on a Samsumg Galaxy S7 (Samsung GEC, 26, Sangil-ro 6-gil, Gagdong-gu, Seoul, Korea), app that estimates BP by applying the fingertip to the mobile camera	General population	OPTIBP MOBILE APPLICATION passed

AAMI, Association for the Advancement of Medical Instrumentation; ABPM, ambulatory blood pressure monitoring; ANSI, American National Standards Institute; BHS, British Hypertension Society; BP, blood pressure; ESH-IP 2010, European Society of Hypertension International Protocol revision 2010; HBPM, home blood pressure monitoring; ISO, International Organization for Standardization; KIGGS, German Health Interview and Examination Survey for Children and Adolescents 2013.

## Supplementary Materials

The following supporting information can be downloaded at: https://www.mdpi.com/article/10.3390/jpm13010009/s1. Scale S1: Scale for the Assessment of Narrative Review Articles (SANRA).
